# Clinical interventions, implementation interventions, and the potential greyness in between -a discussion paper

**DOI:** 10.1186/s12913-016-1958-5

**Published:** 2017-01-07

**Authors:** Ann Catrine Eldh, Joan Almost, Kara DeCorby-Watson, Wendy Gifford, Gill Harvey, Henna Hasson, Deborah Kenny, Sheila Moodie, Lars Wallin, Jennifer Yost

**Affiliations:** 1 School of Education, Health and Social Studies, Dalarna University, FALUN, Sweden; 2 Department of Public Health and Caring Sciences, Uppsala University, UPPSALA, Sweden; 3 Department of Medical and Health Sciences, Linköping University, SE-581 83 LINKÖPING, Sweden; 4 Queens University, KINGSTON, Canada; 5 Public Health Ontario, Toronto, Canada; 6 University of Ottawa, Ottawa, Canada; 7 University of Adelaide, Adelaide, Australia; 8 University of Manchester, Manchester, United Kingdom; 9 Medical Management Centre, Karolinska Institutet, Stockholm, Sweden; 10 Centre for Epidemiology and Community Medicine, Stockholm County Council, Stockholm, Sweden; 11 University of Colorado, Colorado Springs, Colorado, USA; 12 Western University, London, Canada; 13 Department of Neurobiology, Care Sciences and Society, Division of Nursing, Karolinska Institutet, Stockholm, Sweden; 14 Department of Health and Care Sciences, The Sahlgrenska Academy, University of Gothenburg, Gothenburg, Sweden; 15 School of Nursing, McMaster University, Hamilton, Canada

**Keywords:** Concept, Implementation, Implementation Science, Intervention, Knowledge Translation

## Abstract

**Background:**

There is increasing awareness that regardless of the proven value of clinical interventions, the use of effective strategies to implement such interventions into clinical practice is necessary to ensure that patients receive the benefits. However, there is often confusion between what is the clinical intervention and what is the implementation intervention. This may be caused by a lack of conceptual clarity between ‘intervention’ and ‘implementation’, yet at other times by ambiguity in application. We suggest that both the scientific and the clinical communities would benefit from greater clarity; therefore, in this paper, we address the concepts of intervention and implementation, primarily as in clinical interventions and implementation interventions, and explore the grey area in between.

**Discussion:**

To begin, we consider the similarities, differences and potential greyness between clinical interventions and implementation interventions through an overview of concepts. This is illustrated with reference to two examples of clinical interventions and implementation intervention studies, including the potential ambiguity in between. We then discuss strategies to explore the hybridity of clinical-implementation intervention studies, including the role of theories, frameworks, models, and reporting guidelines that can be applied to help clarify the clinical and implementation intervention, respectively.

**Conclusion:**

Semantics provide opportunities for improved precision in depicting what is ‘intervention’ and what is ‘implementation’ in health care research. Further, attention to study design, the use of theory, and adoption of reporting guidelines can assist in distinguishing between the clinical intervention and the implementation intervention. However, certain aspects may remain unclear in analyses of hybrid studies of clinical and implementation interventions. Recognizing this potential greyness can inform further discourse.

## Background

There is increasing global awareness among scientists, healthcare professionals, and decision-makers, that regardless of how valuable clinical interventions may be, they scarcely implement themselves. Rather, the use of effective strategies to implement evidence-based clinical interventions into practice is necessary to ensure that patients receive the benefits. Thus, implementation has become a priority on a *glocal* level, that is, both globally and locally [1]. Accordingly, there is a growing interest in implementation science in healthcare [2].

Implementation science is suggested to be “the scientific study of methods to promote the uptake of research findings” [3] (page 1). The increasing focus on implementation influences not only implementation science but other related scientific areas, such as research on clinical interventions. (Throughout this paper, the term ‘clinical’ conveys health care practice, directed to patients, health care professionals, or both.) While there are similarities between the scientific traditions of clinical research and implementation science, there are also disparities: theoretical and methodological standpoints and approaches may differ, influencing what researchers and health professionals consider important and customary. Clinical intervention trials were typically conducted prior to an implementation intervention study (if any); however, there are now an increasing number of studies comprising both clinical and implementation interventions, applying a so-called ‘hybrid’ design [4]. Such hybrid designs may increase the potential ambiguity in relation to what comprises the clinical intervention and the implementation intervention.

This ambiguity may result from inaccuracy in the application of terms. Further, there may be conceptual as well as concrete grey zones in between a clinical intervention and an implementation intervention. We argue that both health care science and health care practice would benefit from further clarity on the matter of clinical interventions in relation to implementation interventions; teasing out similarities and differences and recognizing the grey areas in between could advance the scientific as well as the clinical discourse. In this discussion paper, we impart our experiences as implementation scientists in Australia, Canada, Sweden, United Kingdom and the United States of America to examine and discuss this issue; we address the concepts of intervention and implementation, primarily as in clinical interventions and implementation interventions, and explore the potential greyness in between, discussing particular approaches that could help to elucidate the greyness.

## Main text

### The suggested greyness: when and why it occurs, and how does it show?

Sometimes, the differentiation of a clinical intervention and an implementation intervention in a health care study is unambiguous - at other times, there is vagueness as to what is what. Ambiguity can transpire in various forms, and different degrees. At times, the greyness may be a result of lack of conceptual precision; in such case, researchers and clinicians can be aided by semantic definitions available. However, as this discussion will demonstrate, there is a potential greyness caused by the ties between a clinical intervention and an implementation intervention. At times these ties are possible to reconcile and it is possible to determine whether the outcomes are related to the clinical intervention or the implementation intervention. At other times, there is a lingering greyness between a clinical and an implementation intervention, originating from the design of the study, the theoretical or scientific propositions, or from the process of performing or reporting the study. Thus, there may be ambiguity in relation to the effect of the clinical intervention, the implementation intervention, or either one or both in a particular context. The researchers can usually recognize the greyness during or after a study; however, it may be others who question the lack of clarity over what is the clinical intervention and the implementation intervention. This greyness is often presented as an ambiguity if it is a question of the clinical intervention and the implementation intervention, the clinical intervention or the implementation intervention, or the clinical intervention versus the implementation intervention. In order to forward this issue in health care research, we consider how attention to issues such as study design, theories, frameworks, models, and reporting guidelines can be applied to aid understanding.

### Defining the concepts of intervention and implementation

As a backdrop to the discussion, a semantic inquiry illustrates the congruence and dissonance, respectively, between the concepts ‘intervention’ and ‘implementation’ [5]. To begin, the concepts have different origins: intervention is an inflection of ‘intervene’, originating from the Latin connotation ‘to come in between’, and implementation is an inflection of ‘implement’; the latter with its origin in Late Latin:’action of filling up’, which later became’to employ’ [6, 7].

As depicted in Tables 1 and 2, the terms resemble one another yet can be considered discrete; while ‘intervention’ indicates involving, in order to improve or help a situation, ‘implementation’ implies carrying something out, or putting (something) into action. Thus, from a semantic perspective, intervention and implementation could be used interchangeably, considering that implementation is itself an intervention. However, an intervention is not implementation per se; even if the noun is specified by adding ‘clinical’ (as in “clinical intervention”), it does not necessarily imply that intervention equates to implementation. Whilst intervention and implementation are not synonyms, from a semantic perspective there are similarities and potential overlaps between a clinical intervention and an implementation intervention.

**Table 1:** Definitions to the verb Intervene, and to the noun Intervention

Term	Exclusive definitions
Intervene	To occur, fall, or come between points of time or events [6, 7]
	To interfere with the outcome or course, esp. of a condition or process [6]
	To come in or between things so as to hinder or modify them [7]
	Come in in the course of an action [59]
	To become involved in a situation in order to improve or help it; to exist between two events or places [60]
Intervention	To happen between two times or between other events or activities [61]
	When someone becomes involved in something [62]

**Table 2:** Definitions to the verb Implement, and the noun Implementation

Term	Exclusive definitions
Implement	Carry out, accomplish, especially to give practical effect to and ensure of actual fulfilment by concrete measures; to provide instrument or means of expression for [6]
	A means of achieving and end [7]
	Carry into effect [59]
	To make something that has been officially decided start to happen or be used [60]
	To put a plan or system into operation [61]
	Make an idea, plan, system or law start to work [60, 62]
Implementation	‘Filling up’ [59]

In the health research context, a clinical intervention is described as any intentional action designed to result in an outcome [8]. Thus, clinical interventions establish the magnitude of the effect of an intervention on health related outcomes [4, 9, 10]. This effect can be determined through the conduct of individual studies or knowledge syntheses (for example, systematic reviews), and knowledge tools (e.g. guidelines, decision aids, pathways) informed by a synthesis of the best available evidence [11–13]. Clinical interventions establish effects for specific clinical practices and programs, systems for the delivery of care, and even health related policies or legislation [4, 8]. Therefore, clinical interventions create the research evidence [14], knowledge [11], or, according to Lavis and colleagues [13] the ‘what’ to be implemented. As illustrated in this paper, this can be for example a falls prevention program or stroke rehabilitation guidelines, originating from clinical intervention studies.

In clinical research, the aim is generally to evaluate the efficiency or effectiveness of a specific clinical intervention, whereas implementation science considers the effectiveness of strategies to change behaviours, in line with the evidence of clinical effectiveness [4]. The concept of implementation intervention can be broadened to any type of strategy that is designed to support a clinical intervention [4]. Implementation interventions are usually complex, with methods or techniques designed to change behaviours at organisational, practitioner or patient levels [15, 16] and to enhance the adoption of a clinical intervention [4, 17]. Further, implementation interventions should be underpinned by appropriate theory to avoid overlooking factors that may be important determinants of practice, linking the theory to outcomes and exploring why, or why not, the implementation intervention was effective [18]. Examples of implementation interventions include computer prompts, audit and feedback, internal facilitators, and in-house training [16, 17]. In this paper, the implementation intervention will be illustrated by a leadership program, as a means to support behaviours suggested to facilitate knowledge implementation.

### Clinical intervention and implementation intervention cases

#### Falls prevention — a clinical intervention

There is convincing evidence that multiprofessional health care teams can prevent falls in older people via a three-step clinical intervention: 1) nursing assessment of the individual’s risk of falls at admission to a healthcare facility, 2) conduct of a medication review (to identify and alter therapies influencing balance), and 3) physical training and other activities tailored to the individual’s identified risk factors [19, 20]. With compelling research evidence at hand, these recommendations have been incorporated into clinical practice guidelines [21] and healthcare policies [22]. However, falls are currently the leading cause of death from injury among older people [20, 23]. Further, falls in healthcare environments are largely attributed to a lack of evidence-based care [24, 25], resulting in calls to improve the implementation of falls prevention interventions [26].

#### Stroke rehabilitation — a clinical intervention

For a person affected by stroke with a residual limitation in activities of daily life (ADL), training in ADL in the home setting after discharge is a top priority [27]; the evidence is as credible for this clinical intervention as it is for the commencement of intravenous thrombolytic treatment within 3 h of the stroke onset [28]. Without rehabilitation, there is a major risk for activity impairment, a negative impact on quality of life, and increased need for symptom relief [28]. Most importantly, the ADL training should be performed in the stroke patient’s home. Yet, even with the evidence at hand, the application of rehabilitation recommendations is variable, indicating a gap between what should be done and what patients are provided in terms of evidence-based care [29].

#### An implementation intervention applied to clinical interventions for falls prevention and stroke rehabilitation

The decisions healthcare leaders make about priorities, commitments and support are critical for staff to change clinical practice and adopt new innovations [30–33]. Thus, an implementation intervention that focuses on leadership capacity building is likely to support the successful implementation of evidence-based practice, in this case, falls prevention and stroke rehabilitation practices. Consistent with previous research and theory on leadership for change [31–36], an implementation intervention focusing on leadership could include:

- creating a sense of urgency to address the clinical issue (in this case: falls, and associated risks for injuries and mortality, and stroke rehabilitation practices),
- understanding the gap between current practice and effective practice (e.g.: assessments, medication reviews, and exercise programs, including techniques and frequency) and setting priorities for change,
- developing an implementation plan and choosing interventions that fit with the current context and barriers to change (e.g.: education, audit and feedback, reminders), and
- supporting the implementation visibly and symbolically (e.g.: establishing clear department standards, recognising staff efforts to change).

#### Illustrating the differences and overlaps causing potential greyness between clinical and implementation interventions

While a clinical intervention can sometimes be supplemented with an implementation strategy (implicitly or explicitly), our cases illustrate the twofold interest for the clinical intervention and the implementation intervention [37, 38]. In both studies the primary outcomes were clinical: the impact of the falls prevention program on rates and severities of falls in older people, and the impact of rehabilitation on ADL function. To tease out the effects of the clinical intervention on these outcomes would involve looking at clinical practice of staff prior to, during and after the studies, that is: the conduct of falls risk assessments, medication reviews and exercise programs, and the conduct of (including the location of) stroke rehabilitation programs. By contrast, the outcomes of the implementation intervention (that is, the leadership program, including tools, training and support) would primarily be process related, including the ‘if’, ‘to what extent’, and ‘how’ the intervention participants conducted the leadership and implementation practices recommended to influence staff to conduct falls prevention or stroke rehabilitation practices.

Distinguishing the effects of the clinical intervention and the implementation intervention can be challenging. For example, poor outcomes may result from deficits related to the clinical intervention, such as lack of staff engagement in falls prevention practice or stroke rehabilitation according to guidelines, or the implementation intervention, such as poor attendance at the leadership program. The combination of the clinical intervention and implementation intervention may also trigger processes, influencing intended outcomes or providing unintended consequences. In our examples, this could be attributed to a lack of clinician buy-in for the intervention because it is not contextually appropriate, or because it differs considerably from accepted current practice. The implementation intervention might fail because of the way the leadership program was promoted and delivered. For example, leaders may state that they value the stroke rehabilitation guidelines, but fail to provide concrete application processes for clinicians. Further, as many implementation scientists recognise, the context in which the clinical intervention and implementation intervention are performed is likely to affect both the processes and outcomes [30]. In the implementing falls prevention and stroke rehabilitation studies by way of a leadership program, we found contextual factors influenced processes and outcomes, for example: whether managers volunteered to participate, opportunities to set aside time for reflection, and the everyday collaboration between the first-line manager, staff reporting to them and relationships with more senior management. Further, while the perceived importance of the clinical intervention for the clinical context rendered effects during and after the leadership intervention, so too did the way the program was delivered and by whom. For example, unpacking a lack of commitment to alter leadership behaviours related to a combination of group dynamics in the leadership program, and the health care professionals’ lack of trust in the evidence proposed by a manager.

### Demonstrating combinations of clinical interventions and implementation interventions, and the potential greyness in between

The potential greyness between clinical interventions and implementation interventions has been attended to for some time, with different methods suggested to address ambiguity. For studies evaluating clinical and implementation interventions, the concept of a hybrid design is proposed [4]. With its aim to study both clinical effectiveness and implementation measures, the hybrid design takes a dual focus a priori, proposing three potential design types:

1. primary testing of the effects of a clinical intervention on relevant outcomes while also observing and gathering information on implementation;
2. dual testing of clinical and implementation interventions, and;
3. primary testing of an implementation intervention while also observing and gathering information on the clinical intervention's impact on relevant outcomes [4] (p 220).

**Fig. 1:**
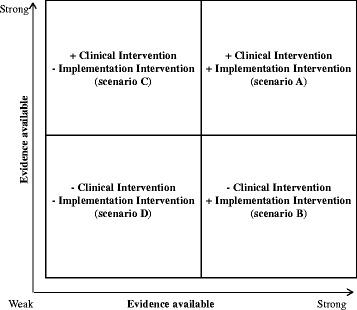
Illustration of aspects to consider in studies including a clinical intervention and an implementation intervention

Thus, a principal issue is to establish whether there is a solid (enough) evidence base. We have found this exercise can be aided by a 2×2 matrix, as suggested in fig. 1. This includes four potential cases (with ‘+‘indicating strong evidence, and ‘-‘weak evidence):

- Scenario A illustrates a well-established evidence base for both the clinical intervention and the implementation intervention. In this case, greyness could occur in relation to what is an effect of the clinical intervention or the implementation intervention, or a combination of the two, in a new context.
- Scenario B illustrates the example of undertaking trials with a primary focus on a clinical intervention, yet combined with an explicit (evidence-based) implementation intervention. In such cases, the effectiveness of the clinical intervention can influence or be influenced by the implementation intervention, and cause greyness. Thus, even with an emphasis on evaluating the clinical intervention, the implementation intervention should be considered, discretely and conjointly: the ‘if’, ‘how’, ‘when’, and ‘why’ it works (or not), in order to investigate how it may affect the clinical intervention.
- Scenario C illustrates a case where the evidence is not settled with regards to the method or strategy to be applied for an implementation intervention while the evidence for the clinical intervention is strong. As the falls prevention and stroke rehabilitation cases elucidate, the primary focus is on testing the relevance of the implementation intervention, while data on the clinical intervention's impact on relevant outcomes are considered.
- Scenario D (indicated by the twin minus-signs of the figure) illustrates cases where neither the evidence base for a clinical intervention is established, nor the evidence with regards to an implementation intervention. While we suggest these conditions are, to the best of our knowledge, rare in health care science, they may occur when a clinical intervention is trialed with an implicit implementation component (that itself is lacking evidence). Such initiatives require particular attention to attribution of observed effects [39].

This 2×2 matrix shares similarities to the hybrid design: a type 1 hybrid coincides with scenario B, and hybrid design three with scenario C (which was the case in our falls prevention and stroke rehabilitation examples). Scenarios A and D correspond more closely to the hybrid design type two. While the hybrid design helps clarify what is what, in terms of clinical and implementation interventions, and processes of data collection and analysis needed to tease out different aspects, we have found that application of the 2×2 matrix helps to further emphasize the evidence aspect relative to clinical and implementation interventions. In the falls prevention and stroke rehabilitation examples, the matrix illustrated the prevailing status in terms of evidence on the clinical intervention, and the need to establish the evidence with regards to the implementation intervention, making communication with health professionals, funding agencies and additional stakeholders more feasible. Even though this only partially addresses the potential greyness in clinical interventions and implementation interventions, these tools could be considered in designing and communicating a hybrid design health care study.

### Using theories, models and frameworks to understand a potential greyness in clinical intervention and implementation interventions

As already noted, establishing a firm theoretical foundation for clinical and implementation interventions can leverage potential synergies and address challenges [40, 41]. There is a substantial body of literature on models and frameworks available, highlighting core concepts and distinguishing features running across multiple implementation frameworks [40, 42–44]. However, it is important to note that, at this point, frameworks are mostly descriptive, rather than predictive or prescriptive [44]. Nevertheless, they offer a useful way to understand the potential greyness that can influence outcomes when clinical interventions and implementation interventions are studied together as hybrids, as presented in the falls prevention and stroke rehabilitation examples. In the case of the falls prevention example, the clinical intervention is based on evidence from clinical trials testing for example the influence of restraints, medications, or balance exercise [20], and for stroke rehabilitation, different types of exercise and team compositions [28]. This demonstrates what we have found to be common, namely that the clinical intervention does not necessarily come with a theory, but with robust evidence (corresponding to the type C scenario in the 2x2), whereas the implementation intervention in our case is emphasized by a careful selection of theories [33].

To inform the testing of implementation strategies, hypotheses based on theory can support the identification of potential influences between the clinical intervention and the implementation intervention a priori, during or after a study with dual foci - and can thus assist in teasing out potential greyness. Yet, many implementation projects do not consider or incorporate theory in a systematic way; Davies et al.’s systematic review of implementation projects and program evaluations found that approximately 23% included a theoretical foundation [18]. Yet, we have found that implementation interventions are reinforced by theories related to the content of the implementation and by process frameworks to guide the logistics of the implementation. If well integrated in the design of the project, theory can guide the direction of the study, and support the analysis and reporting of the process and its outcomes, which is beneficial for both the health care research and clinical communities.

Frameworks and models related to clinical intervention and implementation research have some key differences that can be used to address various variables [43, 44]. At a minimum, being aware of the characteristics will clarify what to look for when selecting frameworks. Generally speaking, clinical intervention frameworks tend to address innovation factors in a comprehensive way, whilst implementation frameworks typically have more stages, address sustainability, and cover more details about the degree and depth of strategies and evaluation [44].

A recently-developed generic implementation framework (GIF) offers an ‘aide-memoire’ to guide consideration of key concepts [44] including: the clinical intervention to be implemented; layers of context; different stages of the implementation process; and factors, strategies, and evaluative methods, and thus key concepts relevant to both clinical and implementation interventions. Inclusion of the core components of the implementation intervention could be helpful in cases where the clinical intervention itself is more clearly defined or has more evidence than the implementation intervention, as in the falls prevention and stroke rehabilitation examples. Researchers can then look to implementation frameworks to ensure comprehensive coverage of aspects informing implementation and evaluation [43]. Similarly, in both the falls prevention and stroke rehabilitation examples, attention was given to details like the pre-implementation activities needed to secure leadership support, as well as evaluation of the implementation intervention. This can assist in detecting and understanding potential links between the clinical intervention and the implementation intervention. Reporting any ambiguity, or ‘greyness’, enhances transparency, providing the research and health care communities with greater opportunity to understand the complexities involved and to recognize needs for further research.

### Teasing out greyness by means of reporting guidelines

Another aid in identifying and reporting greyness between clinical and implementation interventions is consistent reporting of clinical and implementation intervention studies. With challenges of testing and reporting findings from clinical interventions and implementation interventions equally well-documented [45–51], determinants of both healthcare practice and implementation have been outlined [40, 42]. However, reporting remains inconsistent: 97% of implementation intervention studies do not report on uptake, and 69% do not report the setting in which the implementation interventions took place [52].

Significant progress has been made on reporting guidelines for intervention studies over the last 20 years, beginning with the Consolidated Standards of Reporting Trials (CONSORT) [53, 54]. Lately, various extensions of the CONSORT statement have been produced, and there is an international network- Enhancing the Quality and Transparency of Health Research (EQUATOR) Network – with a mission to address the problem of inadequate reporting of research through coordinating and collating research reporting guidelines [54]. The EQUATOR network database provides a useful reference point for guidelines that could be helpful in the reporting of hybrid clinical intervention-implementation intervention studies [40, 42–44]. With a limited number of guidelines to help address the potential greyness, we have examined two reporting guidelines for potential use with hybrid clinical and implementation interventions: the Criteria for Reporting the Development and Evaluation of Complex Interventions in healthcare (CReDECI2) andcbv the Template for Intervention Description and Replication (TIDieR). The CReDECI2 allows for a broad range of study designs and captures elements important to the contextual aspects of implementation [55]. Likewise, TIDieR does not map onto a particular study design but focuses on healthcare interventions, requiring an explanation of theory, context, intermediaries, and outcomes [56]. The latter provides a useful template for illustrating the greyness between clinical and implementation interventions by enabling a clearer picture of the details that distinguish the intervention from others like it, and the distinctions that are important for implementation. For example, TIDieR requires outlining the provider and types of location(s) of the clinical intervention; in the falls prevention example, providers could be nurses, rehabilitation professionals and unregulated staff, and the ‘locations’ residential care homes. By comparison, CReDECI would draw out the core components (for example, stroke rehabilitation), along with the theory underpinning the implementation intervention (in this case the Promoting Action on Research Implementation in Health Services framework and the Ottawa Model of Implementation Leadership) [38]. Further, in the stroke rehabilitation study, the national stroke guidelines were assessed for feasibility and usefulness, and data on context was collected, including for example leadership, culture, and organizational structure, in alignment with the criteria of CReDECI related to facilitators, barriers, and external factors that influence implementation. Had they been reported, further transparency would have been provided on the applicability of a clinical intervention and how the implementation intervention worked in the particular context.

In reporting quality improvement studies in healthcare, the Standards for Quality Improvement Reporting Excellence (SQUIRE 2.0) guideline adds a system-level reporting lens with its link between clinical interventions and outcomes [57]. The SQUIRE 2.0 in parts overlaps with TIDieR and the Standards for Reporting Phase IV Implementation Studies (STaRI). The 35-item checklist of STaRI focuses on defining the intervention: details, core components, and adaptations along with their relationship to treatment outcomes, implementation outcomes, and adverse events [48]. The implementation focus of STaRI covers a breadth of factors including setting, participants, providers, and generalisability and transferability to other contexts, with an explicit distinction between clinical outcomes and implementation outcomes.

Considering the comprehensiveness of reporting guidelines, CReDECI2 and STaRI stand out in terms of the explicit reporting they require with regards to: theory underpinning both the clinical intervention and implementation intervention; outcomes (intervention impact) and process (implementation); context (internal and external), and; level of detail for the intervention, fidelity, costs, and adaptations during the project. While we have applied neither of these guidelines in the clinical intervention and implementation intervention studies presented in this paper, we suggest that both of these reporting tools offer promising routes to tease out potential greyness in clinical and implementation interventions in the future.

### Conceptual, theoretical and practical aspects considered - where does it leave clinical and implementation interventions and the potential greyness in between?

The initial semantic exercise of this discussion proposes clarity of definitions as a primary means to avoid greyness in terms of intervention and implementation. Further, adding appropriate attributes would help distinguish a clinical intervention from an implementation intervention. Our discourse implies that researchers should address both deliberate and unintentional aspects of study processes and outcomes, in order to provide the most thorough illustration possible. This would support health care professionals to better judge whether or not particular findings apply, and scientists to make informed choices. We suggest a simple exercise such as the 2x2 matrix can aid in assessing what is the current state of the evidence (regarding the clinical intervention and the implementation intervention), indicating opportunities and measures required in hybrid design studies. As further illustrated, by means of the clinical interventions and implementation intervention examples, there are designs, theories, frameworks and models that can aid in addressing what is what, and any potential greyness prior to, throughout, and post a hybrid clinical-implementation intervention study. In addition, while there is a call for theoretical foundations to be reported in terms of the clinical intervention and implementation details (see Table 3), we have found no clear, one-size-fits-all approach either to theories, frameworks and models, or reporting guidelines. For the latter we have presented a selection with the proviso that these are available for free at http://www.equator-network.org/[58].

**Table 3:** ᅟ

	Standard for Quality Improvement Reporting Excellence (SQUIRE) [57]	Criteria for Reporting the Development and Evaluation of Complex Interventions in healthcare: revised guideline (CReDECI 2) [55]	Developing Standards for Reporting Phase IV Implementation studies (StaRI) [48]	Better reporting of interventions: template for intervention description and replication (TIDieR) checklist and guide [56]
Overview	18-item checklist for reporting system-level interventions, where methods focused on attributing change to interventions	13-item checklist based on development (4 criteria), feasibility and piloting (1 criteria) and evaluation (8 criteria) of complex interventions	35-item checklist focused on reporting for both intervention impact and implementation process for complex interventions	13-item checklist for intervention completeness and replicability; applies questions: why, what, who, how, where, when and how much, assessing tailoring, modifications, and how well
Checklist content	1. Title – focus on the initiative 2. Abstract – summarize key info 3. Problem description - nature and significance of issue 4. Available knowledge – summary of what is known 5. Rationale – theoretical foundations and assumptions 6. Specific aims – report purpose 7. Context – elements considered key at the outset 8. Intervention(s) – details of intervention and team 9. Study of intervention(s) – approach to assess outcomes 10. Measures – both process and impact measures; methods 11. Analysis – methods for making inferences, assessing variation 12. Ethical considerations – how addressed including formal review, conflicts of interest 13. Results – initial steps, evolution, modifications made, contextual elements, associations, unintended consequences, missing data 14. Summary - key findings and project strengths	1. Theoretical basis for intervention 2. Intervention components (rationale, aims, core functions) 3. Intended interactions between components 4. Details of context’s characteristics and role in intervention 5. Pilot test description and impact 6. Control condition description and rationale 7. Details of the intervention delivery strategy 8. Details of materials and tools used 9. Details of delivery fidelity relative to study protocol 10. Process evaluation -description, theoretical basis 11. Facilitators and barriers revealed as influencers via process evaluation 12. External factors influencing intervention delivery or how it worked 13. Cost or resources for delivery	1. Title and abstract Introduction: 2. description of aspect of care that is the focus 3. rationale 4. evidence and 5. theory underpinning the implementation intervention, 6. study aims and distinction between intervention and implementation Method (criteria 7–22): setting, intervention description (new service), population, randomization, data, and analysis details Results (criteria 23–33): population (participation rates and compliance/attrition), fidelity (including modifications/adaptations), outcomes (process and clinical) Discussion: 34. findings interpreted in context of literature, implications General: 35. regulatory, ethical approval, and other registrations, funding, and conflicts of interest	Brief name: 1. name to describe intervention Why: 2. rationale, theory, or goal of intervention components What: 3. physical or other materials, including where to access 4. procedure/process description, including support activities Who provided: 5. intervention provider (expertise, background, and specific training) How: 6. modes of delivery and whether provided individually or in groups Where: 7. type(s) of location(s) of the intervention, including infrastructure or relevant features When and How Much: 8. frequency and time frame Tailoring: 9. details of personalization or adaptation Modifications: 10. description of changes How well: 11. planned (how adherence or fidelity assessed; strategies to enhance fidelity) 12. actual (if adherence/fidelity assessed, extent of intervention delivery as planned)

## Conclusion

This paper is a reflection on clinical interventions and implementation interventions, and the potential greyness in between. While semantics illustrate similarity, we suggest that the terms are not synonymous. Our falls prevention and stroke rehabilitation cases illustrate potential greyness as to what is a clinical intervention and what is an implementation intervention, with opportunities to tease out some of the ambiguities. We suggest that using appropriate designs, frameworks and models, alongside reporting guidelines that lend themselves to hybrid clinical-implementation intervention studies offers a way to add clarity to the design, conduct and evaluation. This input may be beneficial to scholars and healthcare professionals alike. To conclude, we hope that this paper stimulates further clinical and academic exploration and discourse.

## Abbreviations

ADL: Activities of Daily Life
CONSORT: Consolidated Standards of Reporting Trials
CReDECI: Criteria for Reporting the Development and Evaluation of Complex Interventions in healthcare
EQUATOR: Enhancing the Quality and Transparency of Health Research
SQUIRE: Standards for Quality Improvement Reporting Excellence
STaRI: Standards for Reporting Phase IV Implementation Studies
TIDieR: Template for Intervention Description and Replication
